# The Impact of Age on the Effectiveness of Immune Checkpoint Inhibitors Therapy in Patients with Metastatic Non-Small-Cell Lung Cancer

**DOI:** 10.3390/geriatrics10040085

**Published:** 2025-06-27

**Authors:** Yuliia Moskalenko, Oleksandr Yazykov, Olena Vasylieva, Kateryna Smiian, Tetiana Ivakhniuk, Hanna Budko, Roman Moskalenko

**Affiliations:** 1 Department of Oncology and Radiology, Sumy State University, 40007 Sumy, Ukraine; 2 Department of Surgery, Sumy State University, 40007 Sumy, Ukraine; 3 Department of Pediatric, Sumy State University, 40007 Sumy, Ukraine; 4 Department of Public Health, Sumy State University, 40007 Sumy, Ukraine; 5 Department of Pathology, Sumy State University, 40007 Sumy, Ukraine

**Keywords:** age, non-small-cell lung cancer, immune checkpoint inhibitors, toxicity, treatment duration

## Abstract

The global aging population has led to a growing incidence of malignancies, including metastatic non-small-cell lung cancer (mNSCLC). Immunosenescence may affect the efficacy of immune checkpoint inhibitors (ICIs). The prognostic role of age in ICI-treated mNSCLC remains uncertain. **Objectives**: This study aims to assess whether age independently influences survival, response, and toxicity in mNSCLC patients treated with ICIs, and to examine potential interactions with clinical factors. **Methods**: In this retrospective cohort study, 105 patients with mNSCLC treated with ICIs were enrolled. Patients were stratified into four groups based on age quartiles. Clinical, pathological, and treatment data were collected. Survival outcomes were analyzed using Kaplan–Meier curves, ROC curve and multivariable Cox regression models adjusted for confounders. Interaction and restricted cubic spline analyses were performed to explore age-related effects. The *p* < 0.05 was considered as statistically significant. **Results**: The median age was 60.8 years. Clinical benefit—defined as objective response rate (51.4%) and disease control rate (86.6%)—did not significantly differ across age quartiles (*p* = 0.551 and *p* = 0.257, respectively). Median overall survival also did not differ significantly (*p* = 0.2853). Cox regression and spline modeling demonstrated no independent association between chronological age and all-cause mortality (Model 3: HR = 1.00, 95% CI: 0.95–1.04, *p* = 0.889). However, interaction analyses revealed that poor ECOG performance status (*p* = 0.001), longer duration of ICI treatment (*p* < 0.0001), and low PD-L1 expression (*p* = 0.017) were stronger predictors of mortality in older patients. Age was associated with increased immune-related adverse events and higher Charlson Comorbidity Index scores, suggesting the need for age-specific management strategies. **Conclusions**: Age alone does not predict survival in mNSCLC patients receiving ICIs. However, functional status, treatment duration and PD-L1 expression may modify age-related outcomes.

## 1. Introduction

The risk of developing malignant neoplasms steadily increases with age. In 2050, approximately 6.9 million cancer cases will be registered among individuals over 80 years old [1]. Senescence leads to a decline in the functional capabilities of the human body and accumulation of DNA damage in cells [2]. Older individuals experience changes in various organs and systems, including the immune system. Immunosenescence is a natural phenomenon characterized by a decline in adaptive immunity and an increased risk of malignant tumor development [3]. Interestingly, despite weakening the body’s defense mechanisms, elderly and senile individuals develop a pro-inflammatory status. The precise causes of this condition remain unclear. Several theories have been proposed; one plausible explanation may involve chronic exposure to infectious agents in the context of an aging immune system [4,5].

Immunosenescence is closely linked to involutional changes in the thymus, an immune organ responsible for the development of adaptive immunity. Senescence predominantly affects T cells. Functionally and morphologically defective T lymphocytes fail to ensure reliable antitumor immunity, increasing the risk of cancer [6,7].

Thus, the incidence of cancer increases with the growing elderly population. Treating elderly cancer patients presents significant challenges, particularly for those with metastatic disease requiring systemic therapy (chemotherapy, targeted therapy, and immunotherapy) [8]. Immune checkpoint inhibitors (ICIs) are a promising alternative of chemotherapy with fewer adverse effects, better tolerability, and improved quality of life [9,10]. Based on the theory of immunosenescence, ICIs might be expected to be less effective in older individuals. On the other hand, low-grade chronic inflammation could enhance the response to immunotherapy.

The scientific literature does not provide definitive data about the relation regarding the efficacy of ICIs and age of patients with metastatic non-small-cell lung cancer (mNSCLC). Some authors do not associate aging with decreasing efficacy of ICIs and advocate for immunotherapy regardless of patient age. For example, Yamaguchi et al. [11] found that ICI therapy improved survival in older adult patients, with similar safety and efficacy profiles to younger patients. Conversely, Al-Danakh et al. [12] concluded that senile patients had a poorer response to ICI therapy than younger patients due to immunosenescence and qualitative and quantitative alterations in tumor-infiltrating immune cells. The authors suggested that aging related immune system dysregulation leads to immune cell exhaustion and diminished response to ICI therapy. Consequently, the prognostic significance of age in ICI therapy remains unclear.

Previous large-scale analyses have also yielded inconsistent findings. A meta-analysis by Sun et al. [13] including 2662 patients younger than 65 and 1971 patients aged ≥65 found no significant age-based differences in ICI efficacy. Conversely, Lichtenstein et al. [14] reported significantly shorter progression-free and overall survival among NSCLC patients aged ≥ 80 receiving ICI therapy. Similarly, Xu et al. [15] observed reduced efficacy in patients ≥ 75 years. These contrasting results underscore the need for further age-stratified research. For this study, we defined “older adults” as individuals aged ≥ 70 years, which aligns with established thresholds in geriatric oncology research.

We hypothesized that chronological age alone does not independently predict survival outcomes in patients with metastatic non-small-cell lung cancer treated with immune checkpoint inhibitors. The specific aim of this study was to evaluate the association between age and overall survival, treatment response, and toxicity, while exploring how age interacts with other clinical factors such as ECOG performance status, PD-L1 expression, and treatment duration.

## 2. Materials and Methods

### 2.1. The Ethics Committee

The study was approved by the Commission on Bioethics in Experimental and Clinical Research of the Educational and Scientific Medical Institute of Sumy State University (№ 3/12, date of approval 17 December 2024). All patients who were alive at the start of the study signed an informed consent form.

### 2.2. Patient Population

This retrospective, single-center cohort study included consecutively enrolled 105 mNSCLC patients who received ICI therapy at the Sumy Regional Clinical Oncology Center (Ukraine) between 2016 and 2024. Inclusion criteria were age ≥ 18 years, histologically confirmed NSCLC, metastatic disease, and at least one dose of pembrolizumab or atezolizumab. Exclusion criteria included small-cell lung cancer, NSCLC stages I–III, or the presence of another malignant tumor.

### 2.3. Data Collection

Data on patient sex, age, tumor histology, immunotherapy regimen, therapy line, treatment duration, metastatic sites, immune-related adverse events (irAEs), programmed death-ligand (PD-L) expression, and Easten Cooperative Oncology Group (ECOG) performance status were obtained from medical records. For analysis, only patients with complete data on the variables of interest were included; cases with missing data were excluded. The irAEs were identified through manual review of patients’ medical records and graded according to the Common Terminology Criteria for Adverse Events (CTCAE, version 5). The quantitative assessment of comorbidities was performed using the Charlson Comorbidity Index (CCI) [16]. Information on comorbid conditions was obtained from medical records. The index comprises 19 medical categories, each contributing differently to the risk of mortality. All comorbidities (excluding NSCLC) were considered, and a final score was calculated for each patient. The Charlson Comorbidity Index was adjusted for patient age by adding one point for each decade over 40 years of age.

Tumor response was evaluated using the immune Response Evaluation Criteria in Solid Tumors (iRECIST). Mortality data were retrieved from the cancer registry of Sumy Regional Clinical Oncology Center.

### 2.4. Statistical Analysis

Statistical analysis was performed using Stata V.19.5 (StataCorp., College Station, TX, USA; https://www.stata.com, accessed on 6 May 2025), with *p* < 0.05 considered statistically significant. The age was modeling as a continuous variable. We divided all patients into 4 groups according to age quartiles (34 > Q1 ≤ 57, 58 > Q2 ≤ 61, 62 > Q3 ≤ 66, 67 > Q4 ≤ 78). Categorical variables were expressed as percentages and frequencies. The Shapiro–Wilk test was used to assess normality contribution. Continuous variables across four age quartiles were compared using the Kruskal–Wallis test. Associations between categorical variables were analyzed using the Fisher`s exact test or Pearson’s χ^2^ test, as appropriate.

To analyze the survival of the studied population, we constructed three Cox proportional hazards regression models. The impact of age on the survival of patients with NSCLC receiving ICIs was assessed using hazard ratios (HRs) and 95% confidence intervals (CIs). Model 1 was unadjusted. Model 2 was adjusted for sex, histology, and the presence of metastases in the lungs, pleura, liver, and bones. Model 3 was fully adjusted and included sex, tumor histology, metastatic sites (lung, pleura, liver, bones), treatment line, regimen (monotherapy or combination), immune-related adverse events, ICI treatment duration, ECOG performance status, PD-L1 expression, and CCI.

Overall survival (OS) was defined as the time from the initiation of ICI therapy to death or last follow-up, with right-censoring accounted for in Cox regression and Kaplan–Meier analyses. The duration of follow-up and censoring patterns were comparable across age quartiles.

Objective response rate (ORR) was defined as the proportion of patients achieving complete or partial response. Disease control rate (DCR) was defined as the proportion of patients achieving complete response, partial response, or stable disease. Kaplan–Meier curves were used to visualize survival by age quartiles, with the log-rank test used for group comparisons. Receiver operating characteristic (ROC) curves evaluated the sensitivity and specificity of age in predicting mortality. Restricted cubic splines explored nonlinear associations between age and mortality. Likelihood-ratio tests compared spline and linear models. Subgroup and interaction analyses assessed the modifying effects of clinicopathological variables on age-related outcomes.

## 3. Results

### 3.1. Patient Characteristics

Among the 105 patients with mNSCLC, 89 (84.8%) were male, and 16 (15.2%) were female. The mean patient age was 60.8 years (range: 34–78 years). Table 1 presents the stratified patient characteristics by age quartiles.

A statistically significant association was observed between age quartiles, pleural metastases, irAEs and CCI. Compared to the lowest age quartile, individuals in the highest quartile exhibited a higher incidence of pleural metastases (34.6% vs. 7.4%, respectively; *p* = 0.0001). This finding indicates an age-related difference in the frequency of pleural metastasis.

Immune-related adverse events (irAEs) were also most prevalent in the highest age quartile. In age quartile 1, irAEs were reported in 18.5% of patients with metastatic NSCLC, compared to 50.0% in age quartile 4 (*p* = 0.010). The observed age-related differences in irAEs may have important clinical implications, suggesting the need for age-specific monitoring and management strategies in patients receiving immune checkpoint inhibitors.

Compared to the other age quartiles, the majority of patients in the highest quartile had a Charlson Comorbidity Index (CCI) ≥ 3. In quartile 1, a CCI ≥ 3 was observed in 0.0% of patients, compared to 61.5% in quartile 4 (*p* = 0.0001).

Other factors, such as sex, histological tumor subtype, metastatic sites (lungs, liver, bones), therapy line, and immunotherapy regimen did not show statistically significant differences between the four age quartiles.

### 3.2. Treatment Duration

The median duration of ICI therapy in the studied cohort was 8.0 months (range: 1.0–24.0 months). No significant association was observed between treatment duration and age quartiles (*p* = 0.5976, Table 2).

### 3.3. Treatment Response

Complete response was observed in 6/105 (5.7%) patients, partial response in 48/105 (45.7%), stable disease in 37/105 (35.2%), and progressive disease in 14/105 (13.4%). Consequently, the ORR was 51.4%, and the DCR was 86.6%. No statistically significant association was observed between treatment response and age quartiles (Table 3).

### 3.4. Survival Analysis

Cox proportional hazards regression models were used to assess the association between age and all-cause mortality in patients with metastatic NSCLC receiving ICIs. Age was categorized into quartiles, with the lowest quartile (Q1) serving as the reference group. The findings suggest that increasing age, when stratified into quartiles, is not independently associated with an increased risk of all-cause mortality in this patient population (Table 4).

When modeled as a continuous variable, patient age was not significantly associated with all-cause mortality in any of the Cox regression models (Model 1: HR = 1.00, 95% CI = 0.98–1.03, *p* = 0.603; Model 2: HR = 1.00, 95% CI = 0.98–1.03, *p* = 0.533; Model 3: HR = 1.00, 95% CI = 0.95–1.04, *p* = 0.889). These findings support the earlier results based on age quartiles and confirm that age is not an independent prognostic factor for survival in mNSCLC patients treated with ICIs.

**Figure 1:**
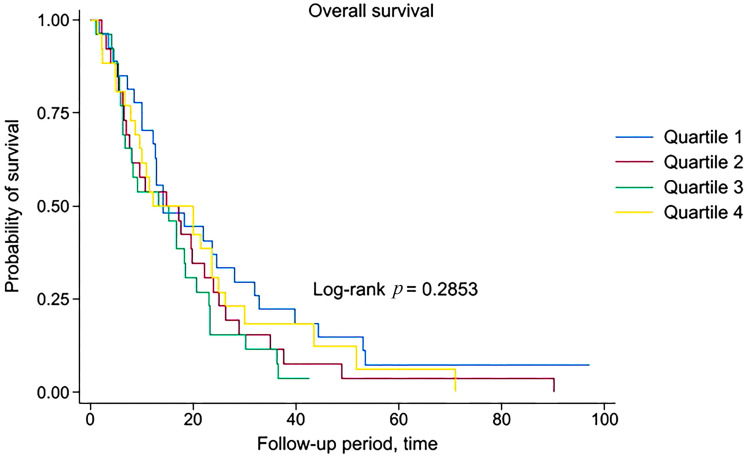
Kaplan–Meier curves for all-cause mortality. The patients are stratified into four groups (Q1, Q2, Q3 and Q4) based on the quartiles of age.

Mortality was recorded in 100/105 (95.2%) patients, including 96/100 (96.0%) deaths due to mNSCLC progression and 4/100 (4.0%) from other causes. The median OS in patients of Quartile 1, Quartile 2, Quartile 3 and Quartile 4 was 14.2, 14.8, 13.4 and 12.1 months, respectively. No statistically significant differences in OS were found among age quartiles (Log-rank *p* = 0.2853). Kaplan–Meier curves demonstrate that survival of patients with mNSCLC is independent of age, which further confirms previously obtained findings (Figure 1).

### 3.5. Restricted Cubic Spline Regression Analysis

**Figure 2:**
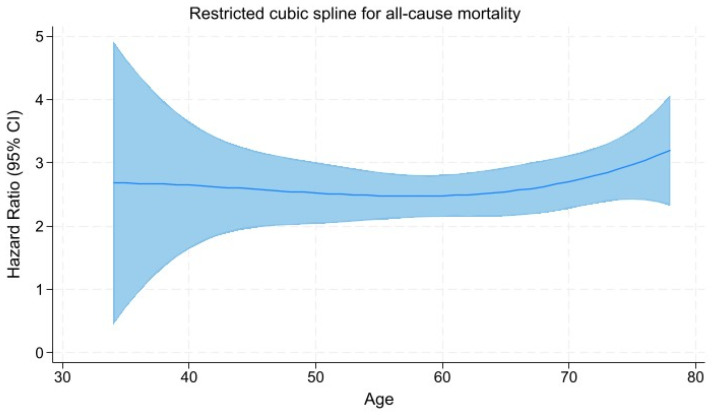
Restricted cubic spline plot showing the nonlinear relationship between age and all-cause mortality in patients with mNSCLC. The solid blue line shows the hazard ratio; the area around the solid line is the 95% CI.

To visualize the association between age of mNSCLC patients and hazard ratio, we used restricted cubic spline regression analysis (Figure 2). The hazard ratios calculated for Models 3, which was adjusted for sex, histology, lung, pleural, liver, and bone metastases, line of therapy, treatment regimen, irAE, duration of ICI therapy, ECOG performance status, PD-L expression, and CCI, were used to construct the splines.

The likelihood-ratio test comparing the spline model with a linear model demonstrated a significant improvement in model fit (χ^2^ = 99.34, *p* < 0.001), indicating a nonlinear relationship between age and hazard of death.

### 3.6. Subgroup Analysis

**Figure 3:**
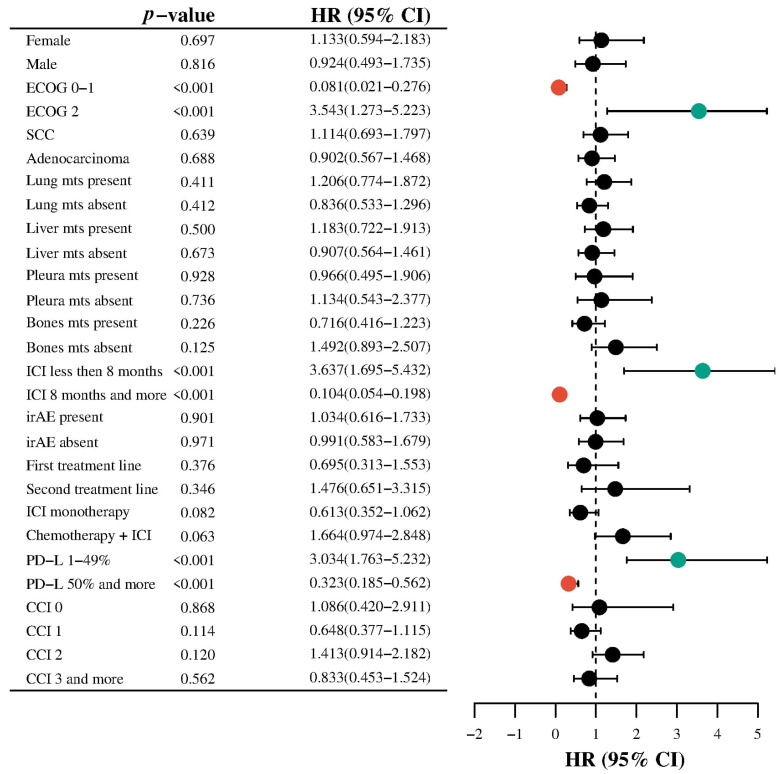
Subgroup analysis for evaluating the association between age and all-cause mortality. Colored circles highlight statistically significant associations (*p* < 0.05): red for protective factors (HR < 1), green for risk factors (HR > 1), and black for non-significant findings.

Subgroup analysis was performed to explore the association between clinicopathological variables and all-cause mortality in patients with metastatic NSCLC treated with ICIs (Figure 3).

ECOG performance status demonstrated a strong association with mortality. Patients with ECOG 0–1 had a significantly reduced hazard of death (HR: 0.081; 95% CI: 0.021–0.276; *p* = 0.0001), whereas those with ECOG 2 had a markedly increased risk (HR: 3.543; 95% CI: 1.273–5.223; *p* = 0.0001).

The duration of ICI therapy was also significantly associated with outcomes. Patients receiving ICI therapy for less than 8 months had a higher risk of mortality (HR: 3.637; 95% CI: 1.695–5.432; *p* = 0.0001), while those with ≥8 months of treatment had a significantly lower risk (HR: 0.104; 95% CI: 0.054–0.198; *p* = 0.0001).

PD-L1 expression levels were another significant predictor. Intermediate expression (1–49%) was associated with increased mortality risk (HR: 3.034; 95% CI: 1.763–5.232; *p* = 0.0001), while high expression (≥50%) was protective (HR: 0.323; 95% CI: 0.185–0.562; *p* = 0.0001). Sex, histologic subtype, and site-specific metastases (lung, liver, pleura, bone) were not significantly associated with mortality. Similarly, irAEs, treatment line, and CCI were not statistically significant predictors in the survival analysis.

While combination ICI with chemotherapy showed a trend toward increased risk (HR: 1.664; 95% CI: 0.974–2.848; *p* = 0.063), and ICI monotherapy showed a trend toward reduced risk (HR: 0.613; 95% CI: 0.352–1.062; *p* = 0.082), these findings did not reach statistical significance.

After that, we performed an interaction analysis between the age of patients with mNSCLC and clinicopathological characteristics that could influence all-cause mortality. The ECOG performance status and age interaction were statistically significant (HR = 1.03, 95% CI: 1.01–1.05, *p* = 0.001), suggesting that the negative prognostic effect of poorer performance status increases with advancing age.

A significant interaction was also observed between PD-L1 expression and age (HR = 0.99, 95% CI: 0.98–1.00, *p* = 0.017). This indicates that the potential protective effect of higher PD-L1 expression on survival becomes more pronounced with increasing age, with a slight decrease in hazard per year of age. A strong and highly significant interaction was found between treatment duration and age (HR = 1.03, 95% CI: 1.02–1.04, *p* < 0.0001), indicating that the association between longer ICI treatment and higher risk of death is amplified in older patients.

These results suggest that age modifies the effect of ECOG performance status, PD-L1 expression, and duration of immunotherapy on overall survival. In particular, older patients appear more vulnerable to poor ECOG status and prolonged ICI exposure, while the beneficial impact of PD-L1 expression may increase with age.

### 3.7. Sensitivity and Specifity Analysis

**Figure 4:**
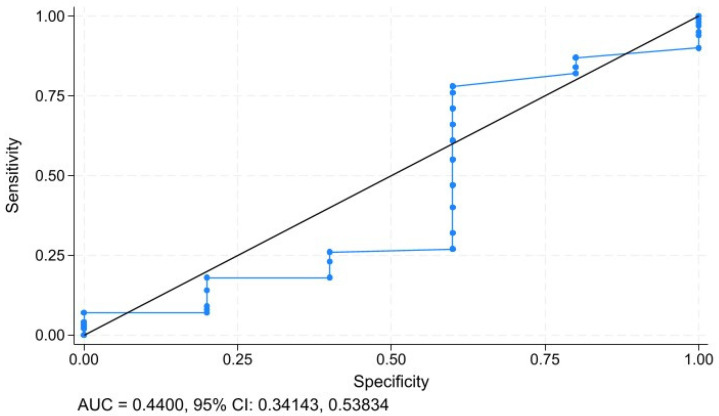
ROC-curve analysis of age and all-cause mortality.

To assess the specificity and sensitivity of age as a prognostic diagnostic tool, we employed ROC curve analysis (Figure 4). The prognostic ability of age in predicting all-cause mortality among patients with metastatic non-small-cell lung cancer (mNSCLC) was demonstrated by an area under the curve (AUC) of 0.4400 (95% CI: 0.34143, 0.53834).

### 3.8. Immune-Related Adverse Events

Patients in the highest age quartile had a higher prevalence of irAEs than those in lower quartiles (*p* = 0.046), particularly hyperthyroidism. However, when analyzing grade 3 or higher toxicity, the results were comparable across all age groups (*p* = 0.936). The comparative analysis of ICI-related toxicity in age quartiles is presented in Table 5.

These findings indicate that while older patients benefited similarly from ICI therapy in terms of survival and tumor response, they experienced a significantly higher incidence of irAEs compared to younger patients (*p* = 0.046). This underscores the importance of proactive toxicity monitoring in this population.

## 4. Discussion

We found that patients in the highest age quartile were more likely to have pleural metastases, irAEs and CCI ≥ 3. However, we did not observe significant differences in the effectiveness of ICIs depending on age. Our findings remained consistent when patient age was analyzed both categorically (in quartiles) and as a continuous variable. In both approaches, age was not significantly associated with overall survival, suggesting that chronological age alone should not be a limiting factor when considering ICI therapy in patients with mNSCLC. Instead, our results suggest that age modifies the effect of ECOG performance status, PD-L1 expression, and duration of immunotherapy on overall survival. We also detected that older patients had a higher rate of irAEs, but this fact did not impact ICI therapy efficacy.

The interaction between tumors and the immune system is highly complex. In peripheral blood, malignant cells undergo apoptosis, releasing antigens that are then processed by antigen-presenting cells. Through antigen-specific T-cell activation, an adaptive immune response is initiated [17]. Upon entering the tumor microenvironment, antigen-specific T cells recognize malignant cells via their receptors, triggering a tumor-specific immune response [18].

Multiple inhibitory and activating factors influence the interaction between tumor and immune cells, with immune checkpoints playing a pivotal role. Programmed cell death protein 1 (PD-1) is a key inhibitory receptor expressed on T cells, regulating their proliferation in response to activation [19]. Immune checkpoint pathways control immune responses at the interface of antigen-presenting cells and T cells, with a particularly critical role in the effector phase within the tumor microenvironment. PD-1 and its ligand (PD-L1) have become major therapeutic targets for ICIs, demonstrating remarkable clinical benefits across various malignancies [20,21,22].

Senescence is associated with functional alterations in the immune system, including a decline in T and B lymphocyte populations, increased regulatory T cells, and reduced antigen-presenting cell activity [23]. The most pronounced changes occur in adaptive immunity, where shifts in memory T-cell pools impair antigen recognition and weaken overall immune responses [24].

These physiological changes raise concerns about the effectiveness of ICI therapy in older patients. However, the prognostic significance of age remains unclear. Arias et al. [25] studied nivolumab in 188 patients aged ≥ 70 years and found no deviation in safety and efficacy compared to the global population. Luciani et al. [26] examined survival outcomes in 86 patients aged ≥ 75 years with locally advanced or metastatic NSCLC treated with ICIs and found no differences in efficacy or toxicity between younger and older patients.

In contrast, Lichtenstein et al. [14] reported age-related differences in ICI efficacy and toxicity. Their retrospective study of 245 NSCLC patients receiving PD-1/PD-L1 inhibitors categorized patients into four age groups: <60, 60–69, 70–79, and ≥80 years. The worst PFS (1.64 months) and OS (3.63 months) were observed in the ≥80-year-old cohort. Regression analysis identified age ≥ 80 years as a negative prognostic factor. Xu et al. [15] similarly reported lower ICI efficacy in patients aged ≥ 75 years. However, the prevalence of non-hematologic irAEs did not differ significantly between groups.

Most studies suggest that ICI toxicity profiles do not significantly differ by age. However, older patients have a higher risk of treatment discontinuation and mortality due to severe irAEs [27,28]. The increased frequency of irAEs in elderly patients suggests a need for age-adapted monitoring protocols. Early recognition and management of these events are critical to maintaining treatment efficacy and reducing toxicity-related morbidity. In our study, patients in the highest age quartile had a higher incidence of irAEs, but this did not affect OS. Similar findings were reported by Ramos et al. [29] and Mebarki et al. [30], who examined ICI efficacy and safety in mNSCLC patients.

We have established that the duration of ICI therapy is significant prognostic factor for OS. The longer treatment period in patients with mNSCLC is associated with better clinical outcomes, underscoring its potential as a marker of treatment efficacy. This finding supports continued ICI therapy in patients who are clinically stable and responsive. On the other hand, based on our previous findings, the identification of a minimum effective ICI treatment duration of at least 8 months holds significant socio-economic implications for patients with mNSCLC. In low-income countries where there is no health insurance coverage and governments do not provide free access to immunotherapy, the full financial burden of treatment often falls on patients’ families. Since ICI therapy is highly expensive, determining the optimal number of ICI infusions required to achieve a beneficial therapeutic outcome becomes a critical issue.

Overall, the benefits of fixed-duration versus continuous ICI therapy in patients with metastatic disease remain a topic of debate. Bogani et al. [31], in a meta-analysis of 57 studies including 22,977 patients, found that prolonged ICI administration (beyond two years) was associated with improved OS compared to fixed-duration treatment. Kim et al. [32] compared clinical outcomes in mNSCLC patients who received ICI therapy for six months versus 24 months and discontinued treatment without disease progression. The authors concluded that patients treated for 24 months had better PFS. However, those who received ICI therapy for only six months also largely achieved a durable response. In our study, no patients received ICI therapy beyond 24 months; however, we observed better treatment outcomes in patients who underwent at least eight months of ICI therapy.

The higher incidence of irAEs observed in older patients may be partly explained by age-related changes in immune function. Immunosenescence, characterized by reduced T-cell diversity and impaired regulatory control, can paradoxically coexist with a chronic pro-inflammatory state known as “inflammaging.” This state of low-grade inflammation may sensitize older individuals to overactivation of the immune system upon ICI exposure [3,5]. Additionally, age-related changes in drug metabolism and clearance may alter the pharmacokinetics of ICIs, potentially leading to greater systemic exposure and toxicity [27]. These mechanisms may underline the increased vulnerability of older adults to irAEs, despite similar therapeutic efficacy.

This study has several important limitations. Its retrospective design may have led to the underrepresentation of more frail older adults, limiting the generalizability of our findings. Moreover, key geriatric factors such as frailty, nutritional status, cognitive function, and social support were not systematically assessed, introducing potential unmeasured confounding. The relatively small sample size, particularly after age stratification into quartiles, may have reduced the power to detect modest differences. Additionally, the absence of tumor molecular profiling limited insights into underlying biological mechanisms. It is also important to note that most patients in our cohort were younger than 80 years; thus, the findings may not extend to the “oldest old” population, who are often underrepresented in clinical studies and may exhibit distinct treatment responses. Finally, the observed absence of survival differences across age groups, despite expected effects of immunosenescence, may reflect residual confounding or cohort selection effects. Future prospective, age-stratified studies that incorporate comprehensive geriatric assessments are needed to better guide treatment decisions in this growing patient population.

## 5. Conclusions

Age is not a primary determinant of ICI therapy efficacy in mNSCLC patients. However, older patients appear more vulnerable to poor ECOG status and prolonged ICI exposure, while the beneficial impact of PD-L1 expression may increase with age. Moreover, older patients require closer monitoring due to the higher incidence of immune-related adverse events. Clinicians should consider age, performance status, PD-L expression, treatment duration and the risk of irAEs when evaluating prognosis and treatment strategy. Regular toxicity assessment and proactive management may improve outcomes, particularly in older patients. Our findings may assist clinicians in making more personalized treatment decisions and facilitate shared decision-making discussions that balance therapeutic benefits with potential risks, especially in elderly individuals with complex health profiles.

## Figures and Tables

**Table 1:** Stratified characteristics of patients by age quartiles.

Variables	Age				*p*-Value (Test)
	Quartile 1, *n* = 27	Quartile 2, *n* = 26	Quartile 3, *n* = 26	Quartile 4, *n* = 26	
Sex, *n* (%)					
Male	21 (77.8)	25 (96.2)	23 (88.5)	20 (76.9)	0.162 (χ^2^)
Female	6 (22.2)	1 (3.8)	3 (11.5)	6 (23.1)	
Histology, *n* (%)					
Adenocarcinoma	17 (63.0)	18 (69.2)	9 (34.6)	14 (53.8)	0.066 (χ^2^)
Squamous cell carcinoma	10 (37.0)	8 (30.8)	17 (65.4)	12 (46.2)	
Metastasis in the lung, *n* (%)					
Absent	12 (44.4)	14 (53.8)	17 (65.4)	13 (50.0)	0.477 (χ^2^)
Present	15 (55.6)	12 (46.2)	9 (34.6)	13 (50.0)	
Metastasis in the pleura, *n* (%)					
Absent	25 (92.6)	26 (100.0)	25 (96.2)	17 (65.4)	0.0001 †
Present	2 (7.4)	0 (0.0)	1 (3.8)	9 (34.6)	
Metastasis in the liver, *n* (%)					
Absent	22 (81.5)	20 (76.9)	17 (65.4)	17 (65.4)	0.447 (χ^2^)
Present	5 (18.5)	6 (23.1)	9 (34.6)	9 (34.6)	
Metastasis in the bones, *n* (%)					
Absent	21 (77.8)	15 (57.7)	16 (61.5)	20 (76.9)	0.269 (χ^2^)
Present	6 (22.2)	11 (42.3)	10 (38.5)	6 (23.1)	
Treatment line, *n* (%)					
First	25 (92.6)	22 (84.6)	23 (88.5)	23 (88.5)	0.801 †
Second	2 (7.4)	4 (15.4)	3 (11.5)	3 (11.5)	
Immunotherapy regimen, *n* (%)					
ICIs monotherapy	10 (37.0)	13 (50.0)	6 (23.1)	9 (34.6)	0.249 (χ^2^)
Chemoimmunotherapy	17 (63.0)	13 (50.0)	20 (76.9)	17 (65.4)	
irAE, *n* (%)					
Absent	22 (81.5)	23 (88.5)	21 (80.8)	13 (50.0)	0.010 †
Present	5 (18.5)	3 (11.5)	5 (19.2)	13 (50.0)	
PD-L1 expression, *n* (%)					
1–49%	22 (81.5)	23 (88.5)	19 (73.1)	18 (69.2)	0.327 †
≥50%	5 (18.5)	3 (11.5)	7 (26.9)	8 (30.8)	
ECOG performance status, *n* (%)					
0–1	26 (96.3)	24 (84.6)	24 (84.6)	26 (100.0)	0.568 †
≥2	1 (3.7)	2 (15.4)	2 (15.4)	0 (0.0)	
CCI, *n* (%)					
0	7 (26.0)	0 (0.0)	0 (0.0)	0 (0.0)	0.0001 †
1	12 (44.4)	11 (42.3)	0 (0.0)	0 (0.0)	
2	8 (29.6)	10 (38.5)	21 (80.8)	10 (38.5)	
≥3	0 (0.0)	5 (19.2)	5 (19.2)	16 (61.5)	

Note: † Fisher’s exact test used; (χ^2^) Pearson’s χ^2^ test used.

**Table 2:** Duration of ICI therapy in mNSCLC patients stratified by age quartiles.

Age Groups	The Median Duration of ICI Treatment (Range), Months	*p*
Quartile 1, *n* = 27	8.1 (1.3–20.0)	0.5976
Quartile 2, *n* = 26	7.6 (1.3–18.7)	
Quartile 3, *n* = 26	7.5 (1.0–18.5)	
Quartile 4, *n* = 26	8.7 (1.0–24.0)	

Note: Kruskal–Wallis test was used.

**Table 3:** Comparison of treatment response among age quartiles.

Treatment Response	Quartile 1, *n* (%)	Quartile 2, *n* (%)	Quartile 3, *n* (%)	Quartile 4, *n* (%)	*p*-Value (Test)
ORR:					
Yes (*n* = 54)	13 (48.1)	11 (42.3)	14 (53.8)	16 (61.5)	0.551 (χ^2^)
No (*n* = 51)	14 (51.9)	15 (57.7)	12 (46.2)	10 (38.5)	
DCR:					
Yes (*n* = 91)	21 (77.8)	23 (88.5)	25 (96.2)	22 (84.6)	0.257 †
No (*n* = 14)	6 (22.2)	3 (11.5)	1 (3.8)	4 (15.4)	

Note: † Fisher’s exact test used; (χ^2^) Pearson’s χ^2^ test used.

**Table 4:** Cox regression analysis on the association between age and all-cause mortality.

	Age for All-Cause Mortality	HR	95% CI	*p*-Value	P for Trend
Model 1	Q1	Reference	–	–	0.370
	Q2	1.19	0.90–1.57	0.208	
	Q3	1.19	0.99–1.44	0.063	
	Q4	1.05	0.91–1.21	0.468	
Model 2	Q1	Reference	–	–	0.577
	Q2	1.24	0.92–1.67	0.143	
	Q3	1.17	0.95–1.43	0.122	
	Q4	1.02	0.87–1.19	0.795	
Model 3	Q1	Reference	–	–	0.716
	Q2	1.19	0.84–1.68	0.317	
	Q3	1.24	0.95–1.62	0.110	
	Q4	1.03	0.83–1.27	0.762	

HR = Hazard ratio; CI = confidence interval. Model 1 was unadjusted. Model 2 was adjusted for sex, histology, and the presence of metastases in the brain, lungs, pleura, liver, and bones. Model 3 was adjusted for sex, histology, lung, pleural, liver, and bone metastases, line of therapy, treatment regimen, irAEs, duration of ICI therapy, ECOG performance status, PD-L expression, and CCI.

**Table 5:** Immune-related adverse events in mNSCLC patients stratified by age quartiles.

irAE	Quartile 1	Quartile 2	Quartile 3	Quartile 4
Pruritus	0 (0.0%)	1 (3.8%)	0 (0.0%)	1 (3.8%)
Hypothyroidism	0 (0.0%)	1 (3.8%)	0 (0.0%)	0 (0.0%)
Hyperthyroidism	0 (0.0%)	1 (3.8%)	1 (3.8%)	4 (15.4%)
Hepatitis	1 (3.7)	0 (0.0%)	0 (0.0%)	1 (2.0%)
Pneumonitis	0 (0.0%)	0 (0.0%)	1 (3.8%)	1 (6.7%)
Nephritis	0 (0.0%)	0 (0.0%)	0 (0.0%)	2 (7.7%)
Colitis	0 (0.0%)	0 (0.0%)	0 (0.0%)	2 (7.7%)
Arthralgia	0 (0.0%)	0 (0.0%)	0 (0.0%)	1 (3.8%)
Myalgia	0 (0.0%)	0 (0.0%)	1 (3.8%)	0 (0.0%)
Bullous pemphigus	1 (3.7%)	0 (0.0%)	0 (0.0%)	0 (0.0%)
Onycholysis	0 (0.0%)	1 (3.8%)	0 (0.0%)	1 (3.8%)
Optic neuritis	0 (0.0%)	0 (0.0%)	1 (3.8%)	0 (0.0%)
Rash	0 (0.0%)	0 (0.0%)	1 (3.8%)	0 (0.0%)
Aseptic bone necrosis	0 (0.0%)	0 (0.0%)	1 (3.8%)	0 (0.0%)
Infusion reaction	0 (0.0%)	0 (0.0%)	0 (0.0%)	2 (7.7%)
Total number of irAE of any grade of toxicity	2 (7.4%)	4 (15.4%)	6 (23.1)	15 (57.7%)
Number of irAE ≥ grade 3 of toxicity	2 (7.4%)	3 (11.5%)	3 (11.5)	1 (3.8%)

## Data Availability

The data that support the findings of this study are not publicly available because they are owned by a third party (Sumy Regional Clinical Oncology Center) and subject to confidentiality restrictions. Any access to the data requires prior approval from the Local Ethics Committee of the institution. Interested researchers may contact the corresponding author to initiate the request process.

## Abbreviations

The following abbreviations are used in this manuscript:

CCI	Charlson Comorbidity Index
ICI	Immune checkpoint inhibitor
mNSCLC	Metastatic non-small-cell lung cancer
